# Relationships between *Legionella* and *Aeromonas* spp. and associated lake bacterial communities across seasonal changes in an anthropogenic eutrophication gradient

**DOI:** 10.1038/s41598-023-43234-3

**Published:** 2023-10-10

**Authors:** Karolina Grabowska-Grucza, Bartosz Kiersztyn

**Affiliations:** Institute of Functional Biology and Ecology, Faculty of Biology, University of Warsaw, Żwirki i Wigury 101, 02-089 Warszawa, Poland

**Keywords:** Environmental sciences, Limnology

## Abstract

Anthropogenic eutrophication of lakes threatens their homeostasis and carries an increased risk of development of potentially pathogenic microorganisms. In this paper we show how eutrophication affects seasonal changes in the taxonomic structure of bacterioplankton and whether these changes are associated with the relative abundance of pathogenic bacteria of the genera *Legionella* and *Aeromonas*. The subject of the study was a unique system of interconnected lakes in northern Poland (Great Masurian Lakes system), characterized by the presence of eutrophic gradient. We found that the taxonomic structure of the bacterial community in eutrophic lakes was significantly season dependent. No such significant seasonal changes were observed in meso-eutrophic lakes. We found that there is a specific taxonomic composition of bacteria associated with the occurrence of *Legionella* spp. The highest positive significant correlations were found for families *Pirellulaceae*, *Mycobacteriaceae* and *Gemmataceae*. The highest negative correlations were found for the families *Sporichthyaceae*, *Flavobacteriaceae*, the uncultured families of class Verrucomicrobia and *Chitinophagaceae*. We used also an Automatic Neural Network model to estimate the relative abundance of *Legionella* spp. based on the relative abundance of dominant bacterial families. In the case of *Aeromonas* spp. we did not find a clear relationship with bacterial communities inhabiting lakes of different trophic state. Our research has shown that anthropogenic eutrophication causes significant changes in the taxonomic composition of lake bacteria and contributes to an increase in the proportion of potentially pathogenic *Legionella* spp.

## Introduction

In recent decades, the human impact on the environment has intensified significantly on a global scale. Increasing urbanization and intensification of agriculture based on the use of artificial fertilizers, together with inappropriate waste management, have contributed to an acceleration of eutrophication in many aquatic ecosystems. Anthropogenic eutrophication significantly accelerates the natural aging of lakes from oligotrophy to eutrophy, with all the consequences of this process^1^.An increase in the concentration of biogenic elements, especially N or P^2^, and dissolved organic compounds in the waters of lakes under high anthropopressure alter the overall water quality^3^ and can cause many unfavorable effects from a human perspective, such as intense blooms of phytoplankton, including toxic cyanobacteria such as *Raphidiopsis* spp. or *Microcystis* spp.^4^. A high concentrations of autochtonous and allochtonous organic matter in eutrophic reservoirs, combined with a global rise in temperature, creates the conditions for an increase in the abundance of heterotrophic bacteria in these reservoirs, including numerous potentially pathogenic species^5^. Bacterial water pollution is one of the greatest concern to the aquatic environment around the world^6^. In previous studies we have found that the presence and abundance of pathogenic bacteria of the genus *Legionella* and *Aeromonas* are associated with water quality parameters related to eutrophication^7^. *Legionella* genus comprises 61 species, among them 22 are responsible for human disease^8^. Usually infection occurs by inhalation of *Legionella*-contaminated aerosols derived from engineered environments such as air conditioning systems, wet cooling towers and water distribution systems^9^. However, the natural habitat of *Legionella* spp. are freshwater reservoirs such as lakes, where potentially pathogenic *Legionella* spp. are widely distributed. One of the transmission-facilitating factors for *Legionella* is the parasitization of various types of protozoa, which can help them survive, multiply, and transport in unfavorable conditions^10^. What is also important, bacteria released from the host are more virulent than microorganisms that omitted intracellular multiplication^11^. *Legionella* is also able to transform into a viable but non-culturable form (dormant cells), which is another strategy to be resistant to bactericidal agents^12^. In most healthy people, *Legionella* infection manifests as a mild febrile illness called Pontiac fever. In case of elderly people with comorbid conditions or people with compromised immune systems, infection with pathogenic strains of *Legionella* takes the form of severe pneumonia with a high mortality rate^9^. In recent years, there has been a worldwide increase in the number of outbreaks of Legionnaires' disease and Pontiac fever. Between 2006 and 2017, data were published on 136 outbreaks of legionellosis of which 115 were Legionnaires' diseases, 4 Pontiac fever and 17 constituted mixed outbreaks^13^. *Legionella* is an opportunistic pathogen that uses the same molecular mechanism that enables it to infect protists to survive phagocytosis by human pulmonary macrophages. The destruction of human macrophages and other antigen presenting cells by *Legionella* is a side effect of the mechanisms that optimize its survival in the environment^14^. *Legionella* is therefore, to a certain extent, “an accidental pathogen”. The case is quite different with pathogenic strains of *Aeromonas* spp. These bacteria have developed a number of pathogenicity mechanisms (membrane components, enzymes and toxins) that allow them to intentionally infect many species of vertebrate organisms^15^. *Aeromonas* spp. are strictly associated with the aquatic environment. Many species are pathogens of fish and cold-blooded animals ^16^. However, over previous decades interest in this genus increased as a human pathogen. Many species of genus *Aeromona*s, with *Aeromonas hydrophila* in the forefront, are the causes of human infections. They are responsible for gastrointestinal, respiratory, skin, soft tissue and urinary tract infections^17^. Pathogenic strains of *Aeromonas* are often the cause of serious infections in people who have suffered water accidents. For example, Hiransuthikul et al.^18^ found that among 777 patients transferred from southern Thailand to 4 hospitals in Bangkok after the December 2004 tsunami, the most common organisms isolated were *Aeromonas* species (145 [22.6%] of 641 isolates from 305 patients).

The previous studies showed that several environmental water quality parameters associated with eutrophication strongly influenced the presence and abundance of *Aeromonas* spp. and *Legionella* spp. in lake waters of the Great Masurian Lakes system^7^. It has also been demonstrated that there is a relationship between the taxonomic composition of bacterial communities and the trophic status of lakes^19,20^. This indicates that advancing anthropogenic eutrophication contributes to a change in the phylogenetic structure of bacterioplankton, which can affect all components of the trophic network of lake ecosystems^19,21^. This can affect the overall biodiversity of microorganisms inhabiting natural waters^21^. Understanding the processes underlying the bacterial biodiversity patterns across space (e.g. trophic gradient) and time (seasonality) is one of the main goals in microbial ecology^22^. *Legionella* and *Aeromonas* belonging to the phylum Gammaproteobacteria are known to require high concentrations of organic matter^23^. It is also known which optimal physico-chemical conditions favor the occurrence of these pathogenes. In our previous work has been found some relation of temperature with *Aeromonas* abundance. In the case of *Legionella*, which have developed a number of mechanisms that allow them to survive in adverse conditions, such as living in biofilms and eukaryotic microorganisms, temperature is less important in assessing their presence and abundance^7^. So far, it has not been reported in detail how *Legionella* and *Aeromonas* interact with other members of the lake bacterial communities. In this work, which is continuation of the previous research, we focus on a different task concerned *Legionella* and *Aeromonas* spp. presence in freshwater ecosystems.

In this paper, we present seasonal changes in the abundance of lake bacteria belonging to the genus *Aeromonas* and *Legionella* against the background of seasonal changes in the taxonomy of aquatic bacteria in the anthropogenic eutrophication gradient. We focus on two main topics. First, we studied how the taxonomic composition of microorganisms from the Bacteria domain changes over time and across a gradient of eutrophication. *We will test the first hypothesis* that the pattern of bacterial taxonomic composition changing over time depends on lake trophic level. Second, we ascend the interaction between *Aeromonas* spp. and *Legionella* spp. and other representatives of the Bacteria domain. By analyzing the relationships of *Aeromonas* spp. and *Legionella* spp. with the coexisting bacterial community, we assess whether there is a specific bacteriocenosis that could be associated with preventing or promoting the spread and development of these pathogens in lake ecosystems. *We will verify the second main research hypothesis,* that there is a specific taxonomic composition of the dominant bacteria in the studied lakes that coexisting with high abundances of representatives of the genera *Aeromonas* and *Legionella*. We also investigate whether, in the case of the Great Masurian Lakes system, it is possible to use the Automated Neural Network (ANN) model to estimate the abundance of *Legionella* spp. and *Aeromonas* spp. based on the frequency of occurrence of the dominant groups of microorganisms associated with these pathogens.

## Results

The surface area of the studied lakes, the geographic coordinates of the sampling sites, and the trophic state index (TSI) values characterizing the lakes are shown in Table 1. More detailed physicochemical data are presented in Table S1 (Supplementary materials). The studied lakes were divided into two groups according to their trophic status: meso-eutrophic (ME, undergo weak anthropopressure), consisting of lakes: Przystań, Mamry, Dargin, Kisajno, Niegocin, Boczne, Śniardwy (TSI to 53) and eutrophic (E, undergo strong anthropopressure), consisting of lakes: Jagodne, Szymoneckie, Szymon, Tałtowisko, Tałty, Ryńskie, Bełdany (TSI above 53).

**Table 1:** Basic data of studied lakes.

Lake	Area [ha]	Coordinates of sampling location	TSI summer	TSI spring	TSI autumn	MEAN TSI	Trophic state
Przystań	115	54.207241, 21.657892	42.18	43.17	41.12	42.16 ± 1.03	ME
Mamry	2 504	54.157234, 21.723144	40.20	44.21	39.40	41.27 ± 2.58	ME
Dargin	3 030	54.145939, 21.731939	42.76	41.86	51.68	45.44 ± 5.43	ME
Kisajno	1 896	54.042000, 21.738594	52.59	43.80	49.98	48.79 ± 4.51	ME
Niegocin	2 600	54.009215, 21.738598	48.47	48.25	54.27	50.33 ± 3.41	ME
Boczne	183	53.967407, 21.758265	50.21	50.32	53.20	51.24 ± 1.70	ME
Jagodne	420	53.945283, 21.721688	58.53	57.95	57.27	57.92 ± 0.63	E
Szymoneckie	523	53.918687, 21.697991	59.27	55.71	56.07	57.02 ± 1.96	E
Szymon	154	53.891046, 21.633682	57.58	58.48	58.08	58.05 ± 0.45	E
Tałtowisko	327	53.880376, 21.560412	56.41	56.97	52.73	55.37 ± 2.31	E
Tałty	1 160	53.813409, 21.567998	59.58	55.33	53.10	55.92 ± 3.16	E
Ryńskie	671	53.935961, 21.544402	59.66	58.83	54.50	57.75 ± 2.86	E
Mikołajskie	498	53.801142, 21.570925	59.14	55.52	53.94	56.20 ± 2.67	E
Bełdany	941	53.686344, 21.578000	60.54	58.76	56.39	58.56 ± 2.08	E
Śniardwy	11 340	53.766621, 21.842127	42.07	42.17	50.53	44.92 ± 4.86	ME

*ME* mesoeutrophic lake; *E* eutrophic lake.

Using Illumina MiSeq sequencing at the family level, we obtained between 2240 and 30 681 OTUs, depending on the sample. A total of 473 different bacterial families were found in the studied lakes. The most abundant bacterial families, those whose average frequency, counted for all the lakes over the entire sampling period, was greater than or equal to 1% belonged to 19 families (Table 2).

**Table 2:** Dominant families whose relative abundance, calculated for all samples over the entire sampling period, was greater than or equal to 1% of the total taxonomic diversity at the family level.

Family	Frequency of dominant 19 Families			
	Mean	Minimum	Maximum	Std.Dev
p__Actinobacteriota;f__Sporichthyaceae	0.22	0.03	0.45	0.11
p__Actinobacteriota;f__Ilumatobacteraceae	0.13	0.03	0.29	0.08
p__Proteobacteria;f__Clade_III	0.06	0.00	0.25	0.06
p__Cyanobacteria;f__Phormidiaceae	0.05	0.00	0.45	0.10
p__Proteobacteria;f__Comamonadaceae	0.05	0.01	0.12	0.03
p__Proteobacteria;f__Burkholderiaceae	0.04	0.01	0.15	0.03
p__Verrucomicrobiota;f__Chthoniobacteraceae	0.03	0.00	0.12	0.03
p__Proteobacteria;f__Methylophilaceae	0.02	0.00	0.03	0.01
p__Actinobacteriota;f__Mycobacteriaceae	0.02	0.00	0.06	0.01
p__Cyanobacteria;f__Prochlorotrichaceae	0.02	0.00	0.10	0.03
p__Planctomycetota;f__Pirellulaceae	0.02	0.00	0.07	0.02
p__Bacteroidota;f__Chitinophagaceae	0.02	0.00	0.04	0.01
p__Cyanobacteria;f__Cyanobiaceae	0.02	0.00	0.08	0.02
p__Planctomycetota;f__Gemmataceae	0.01	0.00	0.06	0.01
p__Chloroflexi;f__SL56_marine_group	0.01	0.00	0.05	0.01
p__Cyanobacteria;f__Microcystaceae	0.01	0.00	0.11	0.02
p__Verrucomicrobiota;f__uncultured	0.01	0.00	0.05	0.01
p__Verrucomicrobiota;f__Methylacidiphilaceae	0.01	0.00	0.08	0.02
p__Bacteroidota;f__Flavobacteriaceae	0.01	0.00	0.07	0.01

**Figure 1:**
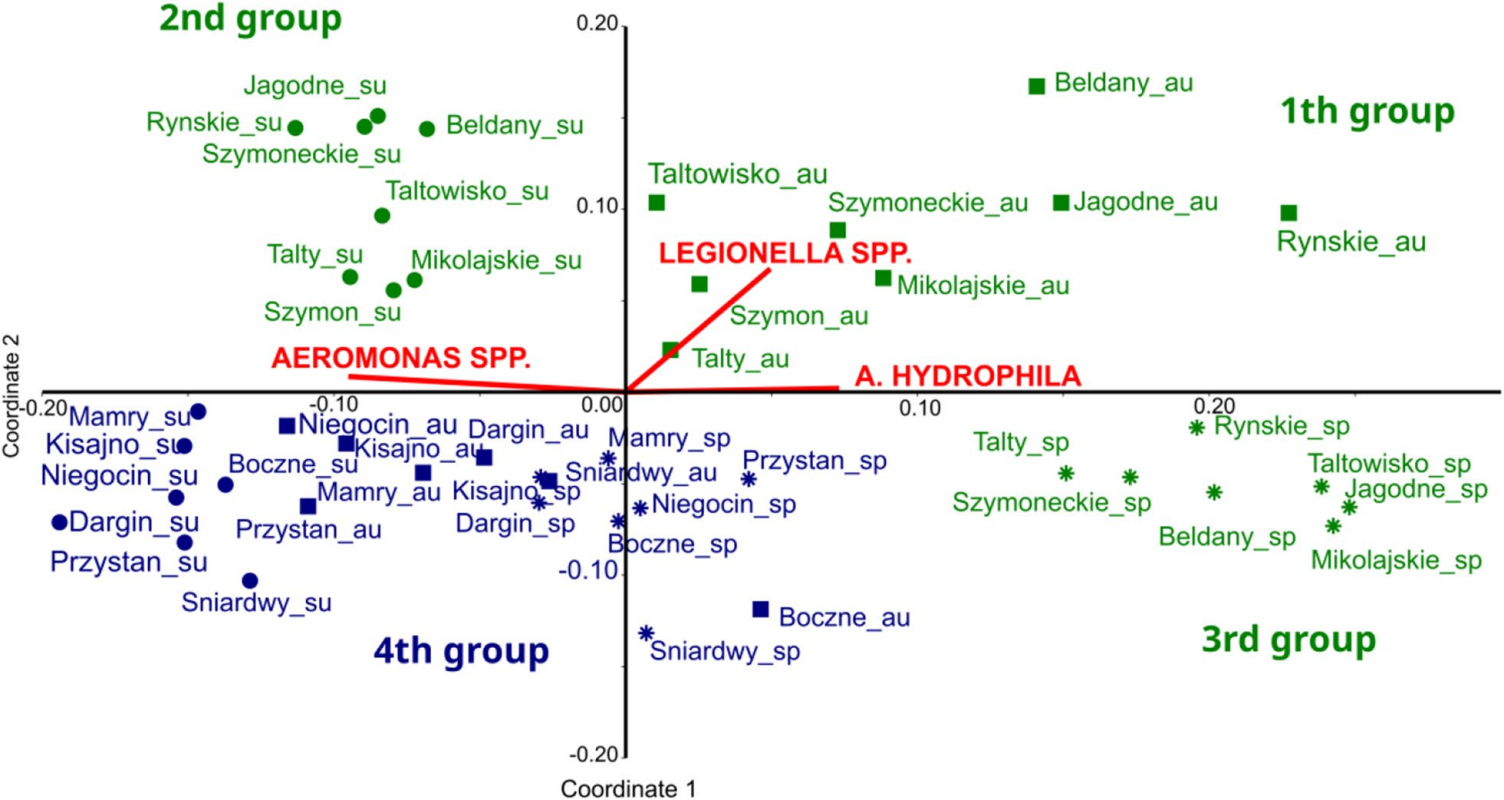
Results of the Bray–Curtis-based NMDS analysis of sequence data, binned by taxonomic assignment to family (stress = 0.13). The whole domain Bacteria was taken into consideration. Green colour indicates Eutrophic lakes (E) and blue meso-eutrophic lakes (ME). The samples taken from lakes in the spring are marked as asterisks, in the summer as dots and in the autumn as squares. The suffixes on the lake names also denote the sampling season: _su—summer, _sp—spring, _au—autumn. Four groups could be found: 1st—samples from eutrophic lakes taken in autumn, 2nd—samples from eutrophic lakes taken in summer, 3rd—samples from eutrophic lakes taken in the spring and 4th –samples from ME lakes taken during the whole sampling period (spring, summer and autumn). The correlation coefficients between *Legionella* spp., *Aeromonas* spp. and *Aeromonas hydrophila* and NMDS scores are presented as red vectors (these variables are not included in the ordination).

In order to analyze the differences between taxonomic composition of bacteria inhabiting studied lakes, we performed Bray–Curtis-based NMDS analyses of sequence data, binned by taxonomic assignment to family. All 473 families were included into analyses. Results are shown on Fig. 1.We have found that, at the family level, the taxonomic composition of bacteria from eutrophic lakes underwent pronounced seasonal changes. The groups of eutrophic lakes studied in spring, summer and autumn were significantly different from each other (Fig. 1, NMDS stress = 0.13, one-way ANOVA results confirming significance of observed differences are presented in Table 3). Such variability has not been observed in meso-eutrophic lakes where the composition of microorganisms from the Bacteria domain was relatively stable trough the seasons compared to eutrophic lakes. The groups of eutrophic lakes (analyzed separately in autumn—1st group, spring—2nd group and summer—3rd group) were significantly different from the group of meso-eutrophic lakes—4th group (one-way ANOVA, R = 0.82, *p* = 0.0001, Table 3). We found that there are a few taxonomic groups of microorganisms that are mainly responsible for the differences between the groups of lakes identified using NMDS analysis. The main families responsible for the observed, significant differences between the studied lake groups (up to ~ 50% of the cumulative contribution) are listed in Table 4, where the results of the Simper tests are shown. For the observed differences between eutrophic lakes sampled during spring and summer (group 3) and meso-eutrophic lakes (group 4), representatives of the phylum Actinobacteriota, Proteobacteria and Verrucomicrobiota were mainly responsible. During autumn, additionally phylum of Cyanobacteria has a large share in explaining the differences between eutrophic and meso-eutrophic lakes. In case of seasonal differences between eutrophic lakes, Cyanobacteria have a constant, high influence. The family *Phormidiaceae* has a particularly strong influence on the observed differences between spring and autumn as well as summer and autumn in eutrophic lakes. It is interesting to note that such seasonal changes did not occur in ME lakes, indicating a relative seasonal stability of bacterial composition in these types of lakes.

**Table 3:** Parwise p coefficient after Bonferroni correction (P Bonferroni correction) and R correlation coefficient of one-way ANOVA test.

P Bonferroni correction	ME su, sp, au	E summer	E spring	E autumn
ME su, sp, au		0.0006	0.0006	0.0006
E summer	0.0006		0.0024	0.0012
E spring	0.0006	0.0024		0.0006
E autumn	0.0006	0.0012	0.0006	

R	ME su, sp, au	E summer	E spring	E autumn
ME su, sp, au		0.7537	0.9343	0.6689
E summer	0.7537		1	0.8316
E spring	0.9343	1		0.8819
E autumn	0.6689	0.8316	0.8819	

The results show that the differences in taxonomic structure of bacterial communities between NMDS-derived groups of samples from eutrophic lakes in spring, summer and fall (E spring—3rd group, E summer—2nd group and E autumn 1st group) and group of meso-eutrophic lakes 4th group (ME su, sp, au) are statistically significant at *p* < 0.05 level.

**Table 4:** SIMPER test results of the contribution and cumulative percentage of families mainly responsible for the observed differences between groups of studied lakes.

	Av. dissim	Contrib. %	Cumulative %
**ME su, sp, au versus E spring**			
p__Actinobacteriota;c__Actinobacteria;o__Frankiales; f__Sporichthyaceae	12.4	20.3	20.3
P__Actinobacteriota;c__Acidimicrobiia;o__Microtrichales; f__Ilumatobacteraceae	8.3	13.6	33.9
p__Proteobacteria;c__Alphaproteobacteria;o__SAR11_clade; f__Clade_III	4.6	7.6	41.5
p__Proteobacteria;c__Gammaproteobacteria;o__Burkholderiales; f__Burkholderiaceae	3.1	5.0	46.5
p__Verrucomicrobiota;c__Verrucomicrobiae;o__Chthoniobacterales;f__Chthoniobacteraceae	2.3	3.7	50.2
**ME su, sp, au versus E summer**			
p__Actinobacteriota;c__Actinobacteria;o__Frankiales; f__Sporichthyaceae	5.8	12.4	12.4
p__Verrucomicrobiota;c__Verrucomicrobiae;o__Chthoniobacterales;f__Chthoniobacteraceae	4.1	8.8	21.3
p__Proteobacteria;c__Alphaproteobacteria;o__SAR11_clade; f__Clade_III	3.3	7.0	28.0
p__Actinobacteriota;c__Acidimicrobiia;o__Microtrichales; f__Ilumatobacteraceae	3.0	6.4	34.0
p__Cyanobacteria;c__Cyanobacteriia;o__Synechococcales; f__Prochlorotrichaceae	2.4	5.2	39.9
p__Verrucomicrobiota;c__Verrucomicrobiae;o__Methylacidiphilales;f__Methylacidiphilaceae	2.4	5.1	45.1
p__Proteobacteria;c__Gammaproteobacteria;o__Burkholderiales; f__Comamonadaceae	1.6	3.4	48.5
**ME su, sp, au versus E autumn**			
p__Cyanobacteria;c__Cyanobacteriia;o__Cyanobacteriales; f__Phormidiaceae	10.2	20.5	20.5
p__Actinobacteriota;c__Actinobacteria;o__Frankiales; f__Sporichthyaceae	7.1	14.2	34.8
p__Actinobacteriota;c__Acidimicrobiia;o__Microtrichales; f__Ilumatobacteraceae	4.1	8.2	42.9
p__Proteobacteria;c__Alphaproteobacteria;o__SAR11_clade; f__Clade_III	3.8	7.6	50.5
**E spring versus E summer**			
p__Actinobacteriota;c__Acidimicrobiia;o__Microtrichales; f__Ilumatobacteraceae	11.1	18.9	18.9
p__Actinobacteriota;c__Actinobacteria;o__Frankiales; f__Sporichthyaceae	6.6	11.2	30.1
p__Proteobacteria;c__Gammaproteobacteria;o__Burkholderiales; f__Burkholderiaceae	2.9	4.9	34.9
p__Proteobacteria;c__Alphaproteobacteria;o__SAR11_clade; f__Clade_III	2.7	4.7	39.6
p__Cyanobacteria;c__Cyanobacteriia;o__Synechococcales; f__Prochlorotrichaceae	2.6	4.5	44.1
p__Verrucomicrobiota;c__Verrucomicrobiae;o__Methylacidiphilales;f__Methylacidiphilaceae	2.5	4.2	48.3
p__Verrucomicrobiota;c__Verrucomicrobiae;o__Chthoniobacterales;f__Chthoniobacteraceae	1.9	3.3	51.6
**E spring versus E autumn**			
p__Cyanobacteria;c__Cyanobacteriia;o__Cyanobacteriales; f__Phormidiaceae	9.6	18.4	18.4
p__Actinobacteriota;c__Actinobacteria;o__Frankiales; f__Sporichthyaceae	5.9	11.4	29.8
p__Actinobacteriota;c__Acidimicrobiia;o__Microtrichales; f__Ilumatobacteraceae	5.1	9.8	39.5
p__Proteobacteria;c__Gammaproteobacteria;o__Burkholderiales; f__Burkholderiaceae	3.5	6.8	46.3
p__Verrucomicrobiota;c__Verrucomicrobiae;o__Chthoniobacterales;f__Chthoniobacteraceae	1.8	3.5	49.8
**E summer versus E autumn**			
p__Cyanobacteria;c__Cyanobacteriia;o__Cyanobacteriales; f__Phormidiaceae	9.8	19.6	19.6
p__Actinobacteriota;c__Acidimicrobiia;o__Microtrichales; f__Ilumatobacteraceae	6.2	12.4	32.0
p__Actinobacteriota;c__Actinobacteria;o__Frankiales; f__Sporichthyaceae	3.9	7.8	39.8
p__Verrucomicrobiota;c__Verrucomicrobiae;o__Chthoniobacterales;f__Chthoniobacteraceae	3.7	7.3	47.1
p__Verrucomicrobiota;c__Verrucomicrobiae;o__Methylacidiphilales;f__Methylacidiphilaceae	2.4	4.8	51.8

*1st* samples from Eutrophic lakes taken in autumn (E autumn), *2nd* samples from eutrophic lakes taken in summer (E summer), *3rd* samples from E lakes taken in spring (E spring) and *4th* samples from ME lakes from whole sampling period (ME su, sp, au). *p* phylum, *c* class, *o* order, *f* family.

In the Figures S2A–C (Supplementary materials) *Legionella* spp., *Aeromonas* spp. and *Aeromonas hydrophila* frequencies for each season are showed. The red vectors in Fig. 1 show the direction of the relationship between the relative abundance of *Legionella* spp., *Aeromonas* spp. and *Aeromonas hydrophila*, and samples grouped by taxonomic similarity. The relationship in case of *Aeromonas hydrophila* and *Aeromonas* spp. were not clearly visible. However, we found a potential link between the abundance of *Legionella* spp. and the specific taxonomic composition that characterizes eutrophic lakes in autumn.

To have a closer look at the relationship between *Legionella* spp. and the representatives of the bacterial families found in the studied lakes, we present tables of Spearman correlations between the frequency of occurrence of *Legionella* spp. and the frequencies of the 19 dominant families. We considered dominant families whose average frequency, calculated for all samples, exceeded 1% (19 families, Table 2) of the total reads at the family level. Data from all samples were taken into account. We found statistically significant (at *p* level < 0.05) negative or positive correlations between the relative abundance of *Legionella* spp. and the frequencies of dominant families (Table 5). The highest positive correlations were for family *Pirellulaceae* (class Planctomycetes, R = 0.69), *Mycobacteriaceae* (class Actinobacteria, R = 0.63) and *Gemmataceae* (class Planctomycetes, R = 0.49). The highest negative correlation were for families *Sporichthyaceae* (class Actinobacteria, R = − 0.79), *Flavobacteriaceae* (class Bacteroidia, R = − 0.68), uncultured family from class Verrucomicrobia (R = − 0.63) and *Chitinophagaceae* (class Bacteroidia, R = − 0.51). Interestingly, the frequency of *Legionella* spp. did not correlate with other representatives of the numerous Proteobacteria phylum, except for a negative correlation with one of the representatives of the Alphaproteobacteria clade (Clade III, R = − 0.50). The Spearman correlations analysis confirm the result of NMDS suggesting strong connection between *Legionella* spp. occurrence and taxonomic composition of coexisting dominant bacterial community.

**Table 5:** Spearman Rank Order Correlations (Bold indicates statistically significant result for *p* < 0.05).

Spearman Rank Order Correlations (Bold indicates statistically significant result for *p* < 0.05)	
Families	*Legionella* spp.
d__Bacteria;p__Actinobacteriota;c__Actinobacteria;o__Frankiales;f__Sporichthyaceae	**− 0.79**
d__Bacteria;p__Bacteroidota;c__Bacteroidia;o__Flavobacteriales;f__Flavobacteriaceae	**− 0.68**
d__Bacteria;p__Verrucomicrobiota;c__Verrucomicrobiae;o__uncultured;f__uncultured	**− 0.63**
d__Bacteria;p__Bacteroidota;c__Bacteroidia;o__Chitinophagales;f__Chitinophagaceae	**− 0.51**
d__Bacteria;p__Proteobacteria;c__Alphaproteobacteria;o__SAR11_clade;f__Clade_III	**− 0.50**
d__Bacteria;p__Cyanobacteria;c__Cyanobacteriia;o__Cyanobacteriales;f__Microcystaceae	**− 0.44**
d__Bacteria;p__Cyanobacteria;c__Cyanobacteriia;o__Synechococcales;f__Cyanobiaceae	**− 0.38**
d__Bacteria;p__Proteobacteria;c__Gammaproteobacteria;o__Burkholderiales;f__Comamonadaceae	− 0.22
d__Bacteria;p__Proteobacteria;c__Gammaproteobacteria;o__Burkholderiales;f__Methylophilaceae	− 0.19
d__Bacteria;p__Chloroflexi;c__SL56_marine_group;o__SL56_marine_group;f__SL56_marine_group	− 0.10
d__Bacteria;p__Cyanobacteria;c__Cyanobacteriia;o__Synechococcales;f__Prochlorotrichaceae	0.26
d__Bacteria;p__Verrucomicrobiota;c__Verrucomicrobiae;o__Methylacidiphilales;f__Methylacidiphilaceae	0.26
d__Bacteria;p__Proteobacteria;c__Gammaproteobacteria;o__Burkholderiales;f__Burkholderiaceae	0.28
d__Bacteria;p__Actinobacteriota;c__Acidimicrobiia;o__Microtrichales;f__Ilumatobacteraceae	**0.30**
d__Bacteria;p__Cyanobacteria;c__Cyanobacteriia;o__Cyanobacteriales;f__Phormidiaceae	**0.46**
d__Bacteria;p__Planctomycetota;c__Planctomycetes;o__Gemmatales;f__Gemmataceae	**0.49**
d__Bacteria;p__Verrucomicrobiota;c__Verrucomicrobiae;o__Chthoniobacterales;f__Chthoniobacteraceae	**0.57**
d__Bacteria;p__Actinobacteriota;c__Actinobacteria;o__Corynebacteriales;f__Mycobacteriaceae	**0.63**
d__Bacteria;p__Planctomycetota;c__Planctomycetes;o__Pirellulales;f__Pirellulaceae	**0.69**

**Figure 2:**
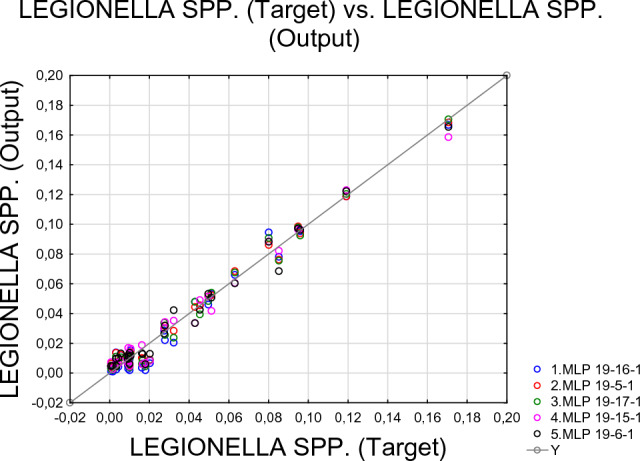
The relationship between percentage share of *Legionella* spp. measured in the samples (Target) vs. percentage share of *Legionella* spp. calculated by ANN models.

Previous analyses suggest that dominant families may interact directly or indirectly with bacteria belonging to *Legionella* spp. In the next step, we examined the possibility of predicting the relative abundance of *Legionella* spp. on the basis of the percentage shares of 19 dominant families present in the studied lakes. In order to create a tool that would allow us to do this, we used the Automated Neural Networks Regression (ANN). The 5 models (3 exponential and 2 logistic) offer a good prediction of the percentages of *Legionella* spp. on the basis of the relative abundance of the representatives of the families being analyzed (Table 6 and Fig. 2). Table 7 shows prediction results based on 5 proposed models compared with observed *Legionella* spp. frequencies. The mean values of 5 regression models of Automated Neural Networks give results close to the observed percentages of *Legionella* spp. in the analyzed lakes. Results of NMDS analyses, nonparametric Spearman correlation analyses and Neuronal Network modelling confirm a strong relationship between *Legionella* spp. occurrence and coexisting dominant bacteria taxonomic composition.

**Table 6:** Summary of active networks. Model 1, 2, 3—exponential, model 4 and 5 - logistic.

Index	Net. name	Training perf	Test perf	Validation perf	Training error	Test error	Validation error	Training algorithm	Error function	Hidden activation	Output activation
1	MLP 19-16-1	0.986,801	0.950.612	0.973,888	0.000.025	0.000.098	0.000.125	BFGS 22	SOS	Identity	Exponential
2	MLP 19-5-1	0.991,756	0.966,570	0.960.950	0.000.014	0.000.065	0.000.156	BFGS 27	SOS	Tanh	Exponential
3	MLP 19-17-1	0.989,857	0.961,745	0.960.096	0.000.018	0.000.079	0.000.107	BFGS 28	SOS	Tanh	Exponential
4	MLP 19-15-1	0.989,088	0.980.711	0.968,014	0.000.018	0.000.038	0.000.044	BFGS 29	SOS	Tanh	Logistic
5	MLP 19-6-1	0.989,643	0.978,100	0.902,219	0.000.017	0.000.048	0.000.307	BFGS 47	SOS	Logistic	Logistic

**Table 7:** Prediction spreadsheet for *Legionella* spp. based on 5 models. The values show the percentage of *Legionella* spp. in the total bacteria number.

Models							
	*LEGIONELLA* SPP. measured	MLP 19-16-1	MLP 19-5-1	MLP 19-17-1	MLP 19-15-1	MLP 19-6-1	Mean from all models
Mamry_su	0.018	0.002	0.006	0.004	0.007	0.006	0.005
Dargin_su	0.001	0.001	0.004	0.003	0.005	0.005	0.003
Kisajno_su	0.004	0.002	0.006	0.004	0.006	0.005	0.005
Niegocin_su	0.001	0.003	0.006	0.005	0.007	0.005	0.005
Jagodne_su	0.171	0.167	0.168	0.170	0.159	0.165	0.166
Szymoneckie_su	0.080	0.095	0.086	0.091	0.088	0.088	0.090
Taltowisko_su	0.085	0.078	0.076	0.076	0.082	0.068	0.076
Talty_su	0.009	0.003	0.010	0.006	0.017	0.012	0.009
Rynskie_su	0.051	0.053	0.052	0.054	0.042	0.051	0.050
Mikolajskie_su	0.016	0.004	0.010	0.006	0.019	0.013	0.010
Beldany_su	0.032	0.020	0.028	0.024	0.035	0.042	0.030
Sniardwy_su	0.002	0.001	0.003	0.002	0.002	0.005	0.003
Przystan_sp	0.010	0.010	0.012	0.011	0.008	0.011	0.010
Mamry_sp	0.017	0.006	0.010	0.008	0.006	0.013	0.009
Kisajno_sp	0.006	0.012	0.012	0.010	0.008	0.013	0.011
Niegocin_sp	0.003	0.014	0.013	0.011	0.007	0.010	0,011
Taltowisko_sp	0.063	0.066	0.068	0.068	0.060	0.060	0.064
Talty_sp	0.050	0.046	0.052	0.048	0.051	0.053	0.050
Mikolajskie_sp	0.045	0.045	0.045	0.039	0.049	0.043	0.044
Beldany_sp	0.119	0.119	0.119	0.120	0.123	0.122	0.121
Przystan_au	0.004	0.004	0.008	0.007	0.006	0.010	0.007
Dargin_au	0.009	0.005	0.007	0.007	0.006	0.009	0.007
Kisajno_au	0.020	0.007	0.009	0.009	0.008	0.013	0.009
Niegocin_au	0.010	0.002	0.005	0.004	0.004	0.006	0.004
Boczne_au	0.011	0.015	0.014	0.012	0.016	0.011	0.014
Jagodne_au	0.096	0.095	0.094	0.092	0.096	0.096	0.095
Taltowisko_au	0.095	0.097	0.098	0.098	0.096	0.097	0.097
Talty_au	0.028	0.028	0,030	0.034	0.030	0.026	0.030
Mikolajskie_au	0.043	0.048	0.044	0.048	0.033	0.033	0.041
Beldany_au	0.028	0.022	0.032	0.026	0.034	0.032	0.029
Sniardwy_au	0.010	0.009	0.011	0.010	0.012	0.013	0.011

The suffixes on the lake names also denote the sampling season: *su* summer, *sp* spring, *au* autumn.

## Discussion

The seasonal and averaged TSIs obtained during our research correspond to the TSI values characterizing the studied lakes over several years^24,25^. Lake trophic state is a proxy for lake productivity, water quality and biological integrity^26^. TSI of lakes from GMLS is closely related to their physical and chemical conditions^27^.The flow of pollutants since the 1970s from the watershed boundary at the level of Lake Niegocin towards the southern lakes and the stronger anthropopressure on the southern lakes compared to the northern lakes create the trophic state gradient of the GML system, which is unique on the European scale. In general, the northern lakes (Przystań, Mamry, Dargin, Kisajno, Niegocin, Boczne) are meso-eutrophic, and the southern lakes (Jagodne, Szymoneckie, Szymon, Tałtowisko, Tałty, Ryńskie, Mikołajskie, Bełdany) are eutrophic. However, one of the southernmost lakes (Śniardwy), which belongs to meso-eutrophic lakes, shows greater similarity in physicochemical and bacterial community diversity to northern, less eutrophic lakes^19,27^.

This article addresses the question of whether the composition of bacterial communities in meso-eutrophic and eutrophic reservoirs shows the same taxonomic stability over the seasons. Based on the results we obtained, the first hypothesis, that the pattern of bacterial taxonomic composition changing over time depends on the trophic level of the lake and is different for lakes under different anthropopressure, has been positively verified. The data showed that the taxonomic composition of bacteria from eutrophic lakes comparing to meso-eutrophic undergoes pronounced seasonal changes. In the NMDS analyses the bacterial communities in the groups of eutrophic lakes in spring, summer and autumn are significantly different from each other (Fig. 1, Table 3). In contrast, bacterial communities in meso-eutrophic lakes sampled at different seasons clustered together what means that the taxonomic composition of the bacterial community in these types of lakes did not undergo pronounced seasonal changes, at least at the family level. Several studies have suggested that the bacterial communities in lake water are altered by different mechanisms after nutrient addition^28^. Eutrophic lakes are subject to dynamic seasonal changes in the concentration and lability of organic matter and the availability of inorganic biogenes. This may result from changes in the abundance and composition of phytoplankton, which excrete dissolved organic products of photosynthesis into the water. Increased nutrient loads are known to directly alter bacterial communities in the lake environment by favouring a group of bacteria with abilities to rapidly consume these sources. This change can be linked to changes in phytoplankton composition^29^. Often, different species of algae are accompanied by different taxonomic bacterial communities, forming a phycosphere that surrounds the algae cells. For example bacteria such as Proteobacteria and Bacteroidetes are more likely to be associated with green algae than other bacterial phylotypes^30^. In addition, eutrophic lakes are usually in an alternating stable state due to, among other factors, internal recycling of phosphorus from sediments. Thus it is impossible to achieve taxonomic stability for many season^31^. This is confirmed by our results, in which groups of eutrophic lakes in each season are different in terms of the taxonomic structure of bacterial communities.

As our results showed for differences between eutrophic lakes in spring and summer (2nd, 3rd groups) and meso-eutrophic lakes group (4th group) were responsible the most representatives of phylum Actinobacteriota. The bacteria from phylum Actinobacteriota are widely distributed in aquatic and terrestrial ecosystems^32^. They are characteristic mainly for oligo- and meso-eutrophic lakes or humic lakes^33^. Actinobacteria have high genomic G + C content and usually contain additional pigments such as melanin. Thanks to this, these bacteria are resistant to UV light^34^, which can easily penetrate the transparent oligotrophic waters. Many Actinobacteria have a life cycle that includes a vegetative and a dormant stage, making them highly resistant to unfavorable environmental conditions and prolonged periods of starvation^33^. This is also in accordance with other research results in which bacteria from the phylum Actinobacteriota, family *Sporichthyaceae* were present in the waters of the lake system in the temperate climate zone (Germany) also in winter^20^.

Another group of bacteria responsible for the differences between meso-eutrophic lakes (4th group) and eutrophic reservoirs, as well as between eutrophic lakes, except for summer-autumn, were Proteobacteria. Proteobacteria are strongly associated with humans, present in various human body tissues, and are one of the most abundant phyla in the human gut microbiome. Many pathogens are also found in this phylum^35^. Within the Proteobacteria phylum *Burkholderiaceae* was responsible for observed differences between spring eutrophic and meso-eutrophic reservoirs, and between eutrophic lakes in spring and the eutrophic lakes analyzed during the rest of the seasons. Bacteria from that family have been reported to manage widespread adaptation to different ecological niches and can perform very diverse metabolic functions, i.e. aromatic compound catabolism and nitrogen fixation^36^. This genus is well-known for its human, animal and plant pathogenic members^37^. In turn, bacteria from order SAR11 (Pelagibacterales) were responsible for the differences between eutrophic lakes in summer and autumn and meso-eutrophic reservoirs. SAR11 are abundant and successful bacterioplankton lineages, which mainly rely on the availability of dissolved organic matter in water^38^. As a result of their genome reduction, they require an unusual range of nutrients, which leads to complex biochemical interactions with other microorganisms. They are found throughout the oceans, but they are typical of most oligotrophic environments. SAR11 bacteria are minimal in size and complexity that is thought to benefit them by lowering the costs of replication and maximizing transport functions that are crucial to competition at very low nutrient concentrations^39^. Verrucomicrobiota phylum is also responsible for the observed differences in bacterial taxonomic composition between meso-eutrophic lakes and eutrophic lakes in spring and summer. The Verrucomicrobiota was also a factor that influenced the observed difference between the eutrophic reservoirs in all the seasons. Other studies have shown that free-living Verrucomicrobiota specialize in fucose and rhamnose consumption during spring algal blooms, especially diatom blooms. Diatoms produce sulfated polysaccharides containing methyl pentoses that are challenging to degrade for bacteria compared to other monomers, implicating these sugars as a potential carbon sink^40^. Another studies showed also that Verrucomicrobiota were identified to be more abundant during the spring season. Their high relative abundance was observed in north temperate lake systems^41^. Verrucomicrobiota seasonality explains its impact on the differences, especially between lakes in spring and during other seasons. In case of seasonal differences between eutrophic lakes, Cyanobacteria had a constant high influence. This confirms the known fact that cyanobacteria are the most abundant phytoplankton organisms linked to eutrophication in freshwater systems^42^. The over-enrichment of surface waters with nutrients, especially nitrogen and phosphorus is frequently a key driver of cyanobacteria blooms. In addition, there is widespread agreement that global warming will continue to promote the spread of cyanobacteria blooms around the world, even in some regions of the world where these phenomena have not previously occurred^43^. Cyanobacteria blooms deteriorates water quality, making it less suitable for recreation, drinking, fishing, etc. and resulting in high economic costs and losses^44^. Some of the cyanobacteria can be toxic to humans and animals, with research showing that the toxic strains may benefit more from warming and eutrophication than the non-toxic ones^45^. In our study within the phylum Cyanobacteria, the family *Phormidiaceae* was mainly responsible for the differences in taxonomic composition observed between lakes. *Phormidiaceae* are known to form cyanobacterial blooms and may produce toxins including anatoxins and microcystins ^46^.The importance of Cyanobacteria for the observed taxonomic changes at the scale of growing seasons is due to the fact that their abundance and taxonomic composition generally change cyclically throughout the year. Cyanobacteria typically dominate in lakes during the summer and fall seasons^47^. In particular, filamentous taxa with relatively slow growth rates, but also slow sedimentation and low susceptibility to grazing by nanoflagellates, and thus a longer growing season, would have a competitive advantage^48^.

In the studied lakes, we observed statistically significant differences between the taxonomic composition of bacteria inhabiting eutrophic lakes subjected to strong anthropopressure and meso-eutrophic lakes. There were also significant differences in the taxonomic composition of bacteria inhabiting eutrophic lakes studied in different seasons. In this context we addressed our second main research question. Could these changes also be associated with changes in the abundance of typical aquatic opportunistic pathogenic bacteria belonging to the genus *Legionella* and *Aeromonas*? We have formulated the second main hypothesis that: there is a specific taxonomic composition of the dominant bacteria in the studied lakes that coexist with high abundances of representatives of the genera *Aeromonas* and *Legionella*. The purpose of verifying this hypothesis was to determine whether there is a taxonomic composition of bacteria that coexists well with *Legionella* and *Aeromonas* spp. The problem is complex because there are several possible interactions. From a preference for the same physicochemical conditions, through interactions between the studied bacteria and various protists and viruses, to competition for resources or alternatively cooperation in their acquisition.

Based on the NMDS analysis (Fig. 1) we found a clear connection between higher *Legionella* spp. abundance and the bacterial communities living in the eutrophic lakes in autumn (1st group). No such clear relationship was found for *Aeromonas* spp. or *Aeromonas hydrophila*. The existence of a relationship between *Legionella* spp. and bacterial communities inhabiting eutrophic lakes was confirmed by Spearman correlation rank test (Table 5). We found several statistically significant positive or negative correlations between the relative abundance of *Legionella* spp. and the relative abundance of 19 dominant families inhabiting the studied lakes on a whole seasons scale. The important families interact (directly or indirectly) with *Legionella* spp. was *Chitinophagacaeae*, negatively correlated with *Legionella* spp. *Chitinophagacaeae* are Gram-negative, mesophilic bacteria. Some species have a dormant stage, which have been called microcysts. They also contain menaquinone type 7, that is sub-type of Vitamin K2, as major respiratory quinone^49,50^. There are reports that menaquinone analogs inhibit the growth of many pathogenic bacteria, like *Streptococcus aureus, Bacillus anthracis, Streptococcus pyogenes, S. agalactiae*, and also *Escherichia coli* and *Salmonella typhimurium*, that belong to Gammaproteobacteria class as *Legionella* spp.^51^. It has been also shown that vitamin K, including menaquinone impact negatively on the production of very potent Shiga toxin 2 (Stx2) by enterohemorrhagic *E. coli*^52^. Although there are no studies on the effect of menaquinone on *Legionella* growth, its negative effects on other pathogenic bacteria from the same class are justification for such further studies. *Sporichthyaceae* had highest negative correlation with *Legionella* spp. abundance (R = − 0.78, *p* < 0.05). It could be the direct effect on *Legionella* spp. because some representatives of *Sporichthyaceae* can control other bacteria spreading secreting antibiotics^53^. However, this assumption requires more detailed research. The strong negative correlation we found also for the SAR11 clade. It could be an example of influence of environmental conditions on bacterial communities. SAR11 clade belongs to Alphaproteobacteria class and is often found in cold environment poor with dissolved organic matter. The importance of SAR11 for ecosystem function in oligotrophic environments may be due to adaptations to low substrate conditions^54^. These conditions are opposite to those favoring the proliferation of *Legionella* spp., which mostly prefer warm waters rich in organic matter^7^.

The case is different for many representatives of the phylum Cyanobacteria. Some cyanobacteria prefer conditions characteristic of many eutrophic environments with high content of nutrient elements and with strong pressure of grazers. The example are representatives of families *Phormidiaceae*—filamentous cyanobacteria, which due to their morphology can avoid grazing by small flagellates. The *Legionella* spp. are able to avoid grazing by hiding inside protist cells. Both organisms are relatively resistant to grazing and can exist in such demanding condition. Grazing-resistant bacterial morphotypes may together follow blooms of heterotrophic flagellates^55^. In support of this assumption, we observed a high positive correlation coefficient (R = 0.5, *p* < 0.05) between the relative abundance of *Legionella* spp. and the relative abundance of *Phormidiaceae*. However, in the case of the phylum Cyanobacteria, we also found a negative correlation between *Legionella* spp. and members of the family *Microcystaceae* (R = − 0.44, *p* < 0.05). The interaction of members of the *Microcystaceae* family with other members of the Bacteria domain may be complex and include allopathic activities related to the release of microcystin into the water by *Microcystis* spp. For example Ramos et al.^56^ observed antimicrobial activity of hexanic extract of *M. aeruginosa* (RST 9501 strain) against *Mycobacterium tuberculosis*. In our study, we observed a positive relationship between representatives of the *Mycobacteriaceae* family and *Legionella* spp. (R = 0.63 *p* < 0.05). We cannot therefore exclude the possibility that interactions between *Legionella* spp. and the *Mycrocystis* family may have the character of negative interactions related to the presence of microcystins.

**Figure 3:**
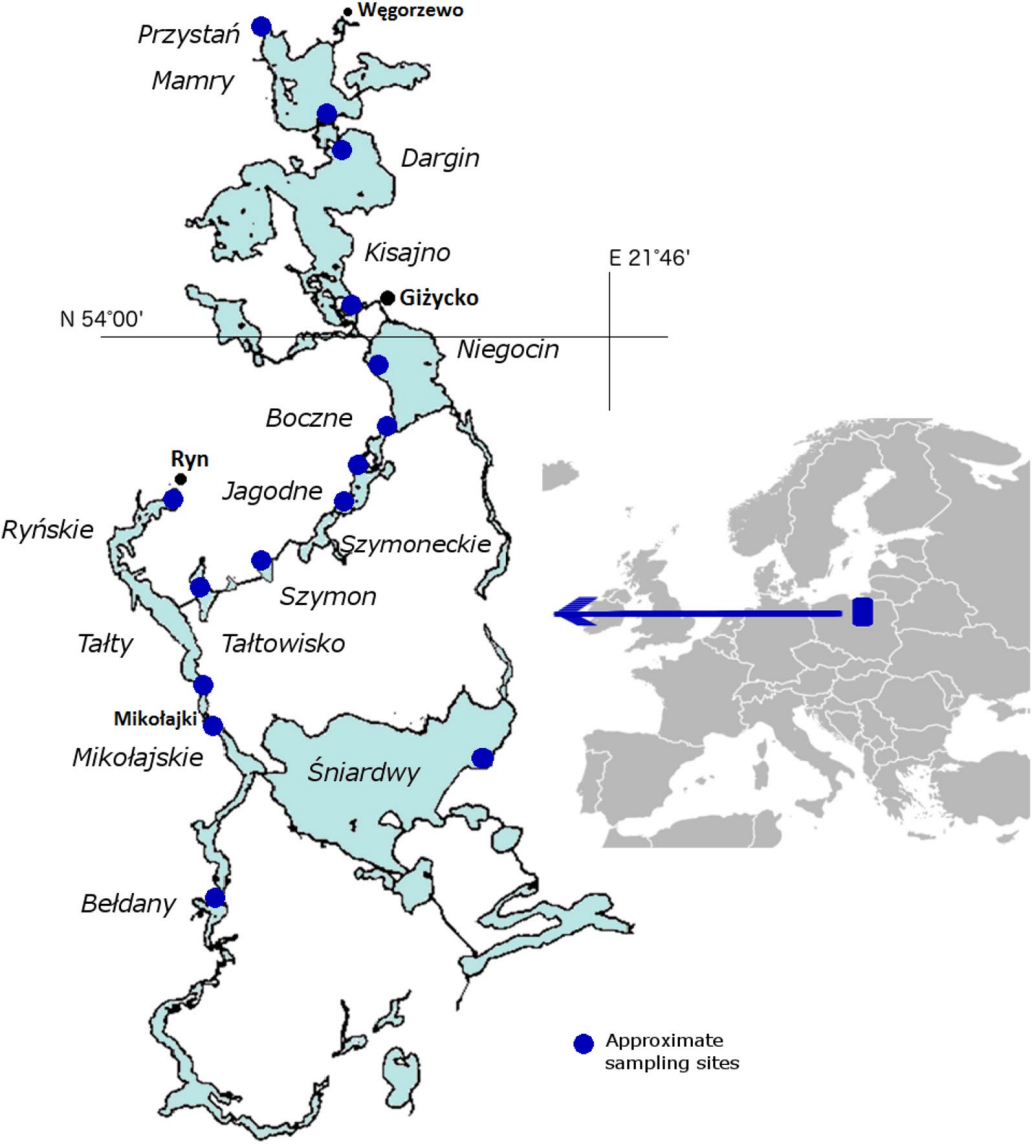
Map of GML (Great Masurian Lakes) system with indicated sampling points.

The observed relationship between the relative abundance of dominant bacterial families and the relative abundance of *Legionella* sp. prompted us to attempt to use an Automated Neural Network to predict *Legionella* spp. occurrence based on the occurrence of 19 bacterial families in the studied lakes. Due to the potential pathogenicity of *Legionella* spp., similar efforts have been made by other researchers. For example, Sinčak et al.^57^ used neural networks to provide an approximation tool to simulate conditions that can prevent legionellosis outbreaks in a water system. We used Automated Neural Networks Regression (ANN) module in TIBCO Statistica®. We found, that 5 model (3 exponential and 2 logistic as output activation) offer a good prediction of *Legionella* spp. share in the whole bacterial community (Fig. 3, Table 6, Table 7). The mean values of 5 regression models of ANN gives results similar to the observed percentage shares of *Legionella* spp. in the analysed lakes. The results confirm a strong relationship between *Legionella* spp. occurrence and coexisting dominant bacteria taxonomic composition.

For *Aeromonas* spp. and *Aeromonas hydrophila*, we found no clear relationships with specific bacterial taxonomic groups. This suggests that there is no simple relationship between *Aeromonas* and the dominant bacterial communities inhabiting lakes of different trophic status. One reason may be that *Aeromonas* spp., as typical pathogenic microorganisms, occur in close association with vertebrates that are their hosts, such as fish or amphibians^16^. Due to improper fisheries management in the eutrophic GML system, fish abundance is low^58^. The lack of hosts may contribute to the decline in *Aeromonas* abundance, despite the potentially favorable living conditions of high summer temperatures and an high concentration of dissolved organic matter.

In summary, anthropogenic eutrophication causes changes in the taxonomic composition of bacteria. The taxonomic structure of bacteria in eutrophic lakes is seasonally variable and differs significantly from the taxonomic structure observed in meso-eutrophic lakes subjected to less anthropopression. The taxonomic composition of bacteria in the waters of meso-eutrophic lakes is subject to less seasonal variation. We also found a statistically significant correlation between the abundance of *Legionella* spp. and the taxonomic composition of accompanying bacteria. This means that there is a taxonomic composition of bacteria where *Legionella* spp. grow and multiply best. In other words, a certain taxonomic composition of bacteria in the studied lakes promotes a high abundance of *Legionella* spp. Such a relationship may be multidimensional and include allopathic or synergistic interactions as well as a preference for certain environmental conditions, both abiotic (e.g. temperature, redox potential) and biotic (e.g. grazers pressure). Understanding of the interactions of individual bacterial species with other coexisting bacteria seems to be a challenge for further research in aquatic microbiology.

## Methods

### The study area

The research area consisted of water reservoirs belonging to the Great Masurian Lakes (GML) system, located in the north-eastern part of Poland. The GML system consists of lakes of different trophic status, connected by natural and artificial channels. It extends for approximately 100 km from north to south. Contractual watershed is located between the lakes Niegocin and Kisajno and forms the border between the northern, meso-eutrophic (Przystań, Mamry, Dargin, Łabap, Kisajno, Niegocin, Boczne) and southern, mostly eutrophic lakes (Jagodne, Szymoneckie, Szymon, Tałtowisko, Ryńskie, Tałty, Mikołajskie, Bełdany and meso-eutrophic Śniardwy) (Fig. 3). This system is unique in Europe. It is located entirely in the temperate climate zone, under similar geological and atmospheric conditions and consists of a moraine and channel-type lakes of postglacial origin. The whole area where the system is located was formed in the late Pleistocene during the Pomorenian phase of the Vistula glaciation and is called the Great Masurian Lake District^59^. It deposits the largest amount of surface water in Poland. The catchment area covers about 3645 km^2^, of which northern part encompases 615 km^2^ and the southern part: 3030 km^2^. The difference in catchment size is one of the reasons for the greater susceptibility of the southern part to euthrophication^24^. Meso-eutrophic lakes in the north differ significantly from eutrophic lakes in the south in terms of physicochemical and biological properties^19^. Detailed information on the examined lakes, including the actual average TSI indicators, are presented in the Table 1. GML system is mainly surrounding by agriculture fields and forests. It is intensively used in tourism, especially in the summer season and has an important impact on the economy in Masurian region. The number of tourists reaches there about one million persons per year^7^.

### Sampling

Water samples were taken from 15 lakes of GML system in summer 2016, spring 2017 and autumn 2017. Sampling sites were located in land–water ecotone zones of the lakes, from 10 to 50 m from the shoreline (depending on the lake and its size). Each sample was collected in triplicate at randomly selected locations within 30 m of the central sampling point. Main sampling sites coordinates are depicted in Table 1. Equal volume of water was taken from every sampling point from three depths: 1, 2 and 3 m using sterile sampling bathymeter. Water from every depth (about 1.67 L) were mixed (v/v) to a volume of 5 L. All samples were then transported to the field laboratory (within 3 h) for further analysis.

### Physico-chemical analyses

At every sampling point the physicochemical parameters (oxygen concentration, turbidity, conductivity, pH) in the whole water column were measured in situ with a YSI 6600 multiparametric probe (Yellow Spring, YSI Inc. USA). The results of physicochemical analyzes are calculated as the average for the entire water column.

The total phosphorus (TP) and orthophosphates (P-PO_4_) concentration were determined spectrophotometrically according to Koroleff^60^ using Shimadzu UV–VIS 1201 spectrophotometer. Chlorophyll *a* (Chl a) concentration was determined according to Arrar and Collins^61^. Extraction was made from 10 mL water samples with 10 mL of 98% acetone. Measuring was carried out with use of a TD-700 fluorometer at 750 nm.

Total nitrogen (TN) was measured using commercially certified Merck-Millipore tests (Spectroquant Nitrogen Total Cell Test, 114,537) and Merck SpectroquantPharo 300 spectrophotometer according to manufacturer’s instructions.

The ammonium (N-NH_4_) content was determined fluorometrically according to Holmes et al.^62^ in a Shimadzu RF 1500 spectrofluorometer.

Dissolved organic carbon (DOC) quantity were measured in water prefiltered through 0.2 µm pore-size polyethersulfone membrane filters (Merck Life Science, Germany).

The trophic state index (TSI) was calculated based of total phosphorus, chlorophyll *a* concentration and Secchi disc visibility, that was measured in situ at every sampling point according to Carlson^63^. Afterthat the TSI values calculated on the basis of these 3 parameters every sampling season were averaged and gaved mean TSI for every sampling site.

### Illumina taxonomy analyses and rtPCR estimation of Aeromonas and Legionella abundance

*DNA was isolated* from microorganisms suspended on 0.2 µm polycarbonate membrane filters (Nuclepore, Whatman, UK) after filtration of 150 mL of each sample water. Isolation was carried out using the GeneMATRIX Soil DNA Purification Kit (EURx, Poland) according to the instruction manual supplied by the manufacturer. Purified DNA was checked for quality and quantity by a Synergy H1 microplate reader (Gen5 software, BioTek, USA) and agarose electrophoresis. The isolated DNA was stored at − 80 °C for further analysis.

*The taxonomic structure of bacterial community was performed using next-generation Illumina sequencing* The V3–V4 hypervariable regions of 16S rRNA genes were sequenced^64^. Amplicons were 459 bp length. PCR amplification was carried out using Q5 Hot Start High-Fidelity 2X Master Mix The reaction mixture included the following components per reaction sample: 12.5 μl of Q5 High-Fidelity 2 × Master Mix, 1.25 μl of 10 μM forward primer, 1.25 μl of 10 μM reverse primer, < 1000 ng of template DNA and nuclease-free water up to a volume of 25 μl. Reaction conditions were as recommended by the manufacturer: 95 °C for 3 min, 25 cycles of 95 °C for 30 s, 55 °C for 30 s, 72 °C for 30 s and, after the last cycle, 72 °C for 5 min. The Bacteria region-specific (341F and 785R)^65^ primers with the Illumina flowcell adapter sequences were forward primer 5′-CGG GNG GCW GCA G-3′ and reverse primer 5′-GAC TAC HVG GGT ATC TAA TCC-3′. The overhang adapter sequences in the locus-specific sequences were forward overhang 5′-TCG TCG GCA GCG TCA GAT GTG TAT AAG AGA CAG-[locus-specific sequence] and reverse overhang 5′-GTC TCG TGG GCT CGG AGA TGT GTA TAA GAG ACA G-[locus-specific sequence]^19^. The Illumina MiSeq sequencer was used with the MiSeq Reagent Kit v2 and 2 × 250 bp protocol. Preliminary automatic data analysis was conducted using the MiSeq system and MiSeq Reporter v2.6 software. This was used to demultiplex the data and to form FASTQ files. Bioinformatic analysis covering the classification of reads was conducted in the QIIME software package using the SILVA reference sequence database (v.138). The abundance of operational taxonomic units (OTUs) at the family level was used for analysis.

*The number of Legionella spp.* was quantified using the commercial and specific mericon Quant *Legionella* spp. Kit (Qiagen, Germany). This kit is ready-to-use system for the detection of specific DNA fragments from *Legionella* spp. in water, animal feed, food and pharmaceutical products using real-time PCR. Using mentioned kit detection and quantification were carried out according to the instruction provided by the manufacturer. The reaction runs were as follows: polymerase activation for 5 min at 95 °C and 40 cycles comprising denaturation for 15 s at 95 °C, annealing and plate read for 23 s at 60 °C and final extension for 10 s at 72 °C.

*Quantification of Aeromonas spp.* was conducted based on the detection of the gyrase B subunit (*gyrB*) gene fragment using primers sequences according to Khan et al.^66^ (forward primer: 5′-CTGAACCAGAACAAGACCCCG-3’, reverse primer: 5′-ATGTTGTTGGTGAAGCAGTA-3′). The amplified product was 130 bp long. Quantification of *A. hydrophila* was conducted using the specific primers for gene encoding the *Aeromonas hydrophila *adhesin (*ahaI*), according to Sebastião et al.^67^: forward primer 5’-GAGAAGGTGACCACCAAGAACA-3′, reverse primer 5′-GAGATGTCAGCCTTGTAGAGCT-3′. The product length was 200 bp. Real-time PCR in case of *Aeromonas* and *A. hydrophila* detection was performed using the iTaq™ Universal SYBR® Green Supermix reaction mixture (Bio-Rad, USA). The reaction mixture per sample contained 5 μL of 1 × concentrated iTaq™ Universal SYBR® Green Supermix, 0.5 μM of each primer, around 50 ng of DNA template and nuclease-free water to the final volume of 10 μL. The standard curve in *Aeromonas* spp. detection and quantification analysis was prepared using quantified genetic material isolated from DNA of *Aeromonas* spp. (accession of sequenced material in Sequence Read Archive: PRJNA523334). For *A. hydrophila *quantification standard curve was prepared from genomic DNA of *A. hydrophila *ATCC 7966 (Minerva Biolabs, Germany). *Aeromonas* and *A. hydrophila* numbers were calculated based on the measured DNA concentration and the genome sequence length. Afterwards a tenfold dilution series, ranging from 10^6^ to 10^0^ cells, were prepared. The reaction run for the quantification of *Aeromonas* spp. and *A. hydrophila* was as follows: polymerase activation for 5 min at 95 °C and 40 cycles comprising denaturation for 5 s at 95 °C and annealing, extension and plate read for 30 s at 60 °C.

The abundance of *Aeromonas* spp. and *Legionella* spp. was related to the total number of bacteria calculated as the total number of bacterial 16S rRNA coding gene copies and expressed as a percentage of the total number of bacteria. To estimate the total bacterial number, the 16S rRNA gene copies of each sample were calculated by real-time PCR using universal forward and reverse primers, EUBF: 5′-TCCTACGGGAGGCAGCAGT-3′ and EUBR: 5′-GGACTACCAGGGTATCTAATCCTGTT-3′^68^.The reaction mixture per sample contained 5 μL of 1 × concentrated iTaq™ Universal SYBR® Green Supermix (Bio-Rad, USA), 0.5 μM of each primer, around 50 ng of DNA template and nuclease-free water to the final volume of 10μL. The standard curve was prepared using quantified genetic material isolated from DNA of *Aeromonas* spp. (accession of sequenced material in Sequence Read Archive: PRJNA523334). The reaction run was as follows: polymerase activation for 5 min at 95 °C and 40 cycles comprising denaturation for 15 s at 95 °C and annealing, extension and plate read for 60 s at 60 °C. Melt curve analysis in all analysis with using iTaq™ Universal SYBR® Green Supermix was conducted over a temperature gradient from 65 to 95 °C at 0.5 °C increments at 5 s per step. All amplification reactions were done in triplicates. A negative control was also applied. The minimal reaction efficiencies were of 90–100% and 0.997 < R2 < 0.999. All reactions were performed using CFX96 Touch™ Real-Time PCR detection system (Bio-Rad, USA).

### Statistical analysis

Nonparametric multidimensional scaling (NMDS) analysis based on Bray–Curtis distances of relative bacterial abundance at the family level was used to group lakes according to taxonomic differences in the bacterial communities that inhabit them. The correlation coefficients between *Legionella* and *Areomonas* spp. abundance and NMDS scores are presented as vectors on NMDS graph. Analyses was done using Past 4 software (Natural History Museum, University of Oslo, https://www.nhm.uio.no/english/research/resources/past/). To test the significance of differences between groups of lakes in particular seasons One-way Anosim test was used (Past 4 software). To determine which families of bacteria were mostly responsible for observed differences between groups of studied lakes, the SIMPER test was used (Past 4 software). Spearman correlations analyses were used to closer assess the relationship between *Legionella* spp., and 19 dominant bacteria families, for which the mean percentage share calculated for whole set of samples was larger than 1%. (TIBCO Software Inc. Statistica, version 13. http://statistica.io.). Automated Neural Network (ANN) was used to create a model that allows prediction of the proportion of *Legionella* spp. in the bacterial community based on the frequency of occurrence of 19 dominant families in the analyzed water samples. We used MLP (multilayer perceptron) network type (min. hidden units 5, max. hidden units 17) based on regression analyses. 5 active neural network was used to predict the percentage of *Legionella* spp. in whole bacterial communities. For the construction of the neural network, the SANN module implemented in Statistica software was used (TIBCO Software Inc. Statistica, version 13. http://statistica.io). For tables and graphs preparation Excel (Microsoft) and GIMP 2.8.22 were used.

### Supplementary Information

Supplementary Information.

## Data Availability

All sequencing reads have been deposited at the NCBI Archive—BioProject: PRJNA509870. The remaining datasets generated and/or analyzed during the current study are available in the manuscript or from the corresponding author on request.
