# Supplementary material for: Relationships between Legionella and Aeromonas spp. and associated lake bacterial communities across seasonal changes in an anthropogenic eutrophication gradient
Source: Sci Rep. 2023 Oct 10;13:17076. doi: 10.1038/s41598-023-43234-3 (PMC10564844; doi:10.1038/s41598-023-43234-3)
Supplement: Supplementary file 1 — Supplementary Information. [file 41598_2023_43234_MOESM1_ESM.pdf]

Relationships between *Legionella* and *Aeromonas* spp. and associated lake bacterial communities across seasonal changes in an anthropogenic eutrophication gradient

Karolina Grabowska-Grucza ✉, Bartosz Kiersztyn

Institute of Functional Biology and Ecology, Faculty of Biology, University of Warsaw, ŻwirkiiWigury 101, 02-089 Warszawa, Poland
✉ e-mail: k.grabowska7@uw.edu.pl

Table S1. Mean values ± standard deviations of the basic physicochemical parameters of the lake water.

Lake	Temp [°C]	Cond [µS cm^{-2}]	Oxygen [mg L^{-1}]	pH	Turbidity [NTU]	SD [m]	Chl a [µg L^{-1}]	TP [µg L^{-1}]	P-PO_4 [µg L^{-1}]	TN [µg L^{-1}]	NH_4^+ [mg L^{-1}]	DOC [mg L^{-1}]
**Przystań_su**	21.35± 0.06	297.00±0 .00	9.77±0.03	8.38±0.01	0.03±0.06	4.10	3.77±0.4 0	15.05±0. 53	3.68±0.35	366.67±57. 74	27.0± 4.62E-03	8.43±0.07
**Mamry_su**	21.68± 0.14	295.00±0 .00	10.05±0.03	8.32±0.05	4.35±5.3	2.50	0.94±1.1 3	15.67±1. 34	3.13±0.21	633.33±11 5.47	17.0± 1.93E-04	8.09±0.07
**Dargin_su**	22.50± 0.02	302.00±0 .00	10.19±0.00	8.50±0.0	0.30±0.14	3.50	3.37±0.5 7	15.67±0. 53	3.48±0.62	533.33±15 2.75	22.0± 2.72E-03	9.11±0.08
**Kisajno_su**	21.64± 1.56	324.00±6 .00	10.27±1.08	8.40±0.09	0.87±0.50	2.10	12.26±1. 69	30.16±1. 54	3.58±0.30	650±70.71	0.019± 1.44E-03	9.23±0.10
**Niegocin_su**	22.38±	370.00±1	10.47±0.26	8.45±0.0	1.20±0.14	2.50	5.29±0.2	26.97±0.	14.26±0.92	600±0.00	19.0± 5.32E-03	11.26±0.03

	0.74	.41					8	64				
**Boczne_su**	20.95±0.01	373.00±0.00	10.05±0.01	8.44±0.06	1.57±0.31	2.70	7.93±0.15	31.80±1.52	6.17±0.46	433.33±57.74	15.0±9.00E-02	11.12±0.30
**Jagodne_su**	22.03±0.03	354.00±0.00	10.08±0.08	8.44±0.0	3.77±0.11	1.10	23.23±1.56	35.19±0.18	3.73±0.21	700±100.00	12.0±1.06E-02	11.99±0.08
**Szymoneckie_su**	21.65±0.01	354.00±0.00	9.55±0.01	8.42±0.01	3.60±0.28	1.10	24.07±0.42	40.13±0.36	3.43±0.21	700±100.00	11.0±3.44E-03	10.08±0.05
**Szymon_su**	20.87±0.01	354.50±0.71	7.61±0.03	8.02±0.01	2.95±0.35	1.20	18.11±0.64	37.35±1.25	3.38±0.46	666.67±152.75	15.0±4.37E-02	10.28±0.03
**Tałtowisko_su**	21.48±0.91	361.00±5.20	9.82±0.16	8.31±0.04	1.90±0.1	1.30	19.05±0.40	30.67±0.71	2.78±0.17	866.67±152.75	11.0±3.78E-04	11.52±0.11
**Tałty_su**	22.66±0.07	306.33±0.58	11.52±0.50	8.54±0.03	6.17±0.38	1.00	12.30±5.54	58.32±0.64	4.48±0.0	800±57.74	11.0±3.49E-03	9.05±0.07
**Ryńskie_su**	22.59±0.11	309,33±0.58	9.90±0.04	8.40±0.0	4.23±0.06	0.95	20.99±0.68	43.42±0.82	3.38±0.17	633.33±0.00	11.0±3.12E-03	9.50±0.02
**Mikołajskie_su**	21.72±0.86	310.33±5.00	10.12±0.94	8.40±0.11	4.80±3.0	1.25	20.86±3.00	48.86±0.36	4.48±0.30	700±100.00	10.0±7.17E-03	9.15±0.11
**Bełdany_su**	23.50±0.11	256.00±0.00	12.65±0.05	8.63±0.01	7.03±0.15	0.95	23.74±0.93	45.57±1.41	3.58±0.30	1000±100.00	10.0±3.49E-03	7.55±0.07

**Śniardwy_su**	23.58±0.13	291.36±1.43	13.58±0.4	8.46±0.02	0.45±0.11	3.00	2.05±1.58	16.28±0.31	3.78±0.17	700±0.00	16.0± 3.46E-03	8.02±0.05

**Lake**	**Temp [°C]**	**Cond [µS cm^{-2}]**	**Oxygen [mg L^{-1}]**	**pH**	**Turbidity [NTU]**	**SD [m]**	**Chl a [µg L^{-1}]**	**TP [µg L^{-1}]**	**P-PO$_4$ [µg L^{-1}]**	**TN [µg L^{-1}]**	**NH$_4^+$ [mg L^{-1}]**	**DOC [mg L^{-1}]**
**Przystań_sp**	11.53±0.16	307.33±0.58	14.04±0.05	8.72±0.06	0.0±0.0	3.5	4.69±1.13	13.61±1.05	4.17±0.30	533.33±115.47	0.014±0.0	7.93±0.05
**Mamry_sp**	9.18±0.21	307.5±0.71	13.98±0.03	8.48±0.01	0.0±0.0	2.5	3.99±0.88	13.51±0.46	2.18±0.46	366.67±57.74	0.021±0.001	7.88±0.13
**Dargin_sp**	11.12±1.43	314±1.41	13.35±0.27	8.55±0.01	0.0±0.0	2.5	1.94±0.08	13.51±1.48	9.0±0.35	350.0±±70.71	0.036±0.0	8.47±0.03
**Kisajno_sp**	11.55±1.03	318.33±0.58	12.78±0.21	8.55±0.01	0.0±0.0	3.1	3.31±0.22	17.4±1.54	2.50±0.17	450.0±70.71	0.024±0.001	8.62±0.08
**Niegocin_sp**	13.57±1.39	387.5±0.71	13.61±0.15	8.66±0.01	0.25±0.21	2	3.99±0.10	24.98±0.75	2.18±0.46	700.0±0.0	0.020±0.001	9.63±0.04
**Boczne_sp**	10.57±0.24	386±0.0	12.31±0.09	8.70±0.06	0.33±0.15	2.55	6.79±0.70	34.15±1.9	2.58±0.75	750.0±70.71	0.015±0.001	9.92±0.03
**Jagodne_sp**	10.79±0.28	402±2.0	13.51±0.07	8.69±0.02	1.93±0.06	1.4	22.25±0.66	40.93±1.20	10.52±13.84	700.0±100.0	0.016±0.001	10.51±0.11
**Szymoneckie_sp**	10.32±	391±0.0	13.55±0.01	8.71±0.01	1.55±0.07	1.5	18.95±0.	30.66±1.	2.60±0.34	700.0±141.	0.011±0.001	10.01±0.11

	0.07						30	13		42		
**Tałtowisko_sp**	11.25±0.46	413.33±0.58	15.51±0.14	8.77±0.0	1.83±0.15	1.6	23.13±0.97	37.14±	3.37±0.17	1333.33±115.47	0.013±0.0	10.63±0.03
**Tałty_sp**	9.46±0.10	386.33±0.58	12.95±0.05	8.69±0.02	1.13±0.06	1.7	15.82±1.01	36.34±	2.78±0.17	1066.67±57.74	0.037±0.0	9.36±0.12
**Ryńskie_sp**	8.62±0.10	379.33±0.58	13.48±0.24	8.66±0.01	1.50±0.0	1.6	25.75±1.40	50.8±	3.27±0.30	1066.67±57.74	0.024±0.0	9.11±0.08
**Mikołajskie_sp**	9.40±0.02	364.33±0.58	12.89±0.01	8.62±0.0	1.23±0.06	1.6	16.51±0.56	34.55±	2.60±0.34	600.0±0.0	0.025±0.0	8.40±0.15
**Bełdany_sp**	9.80±0.66	319.33±3.06	12.65±0.45	8.71±0.06	2.27±0.32	1.2	22.59±2.23	41.13±	2.98±0.0	533.33±152.75	0.014±0.0	6.95±0.01
**Śniardwy_sp**	11.53±0.0	332±0.0	12.72±0.0	8.43±0.0	0.0±0.0	3	2.78±0.35	13.51±	2.28±0.17	499.94±0.0	0.008±0.0	7.62±0.06

**Lake**	**Temp [°C]**	**Cond [µS cm^{-2}]**	**Oxygen [mg L^{-1}]**	**pH**	**Turbidity [NTU]**	**SD [m]**	**Chl a [µg L^{-1}]**	**TP [µg L^{-1}]**	**P-PO_4 [µg L^{-1}]**	**TN [µg L^{-1}]**	**NH_4^+ [mg L^{-1}]**	**DOC [mg L^{-1}]**
**Przystań_au**	15.42±0.09	301±0.0	9.71±0.03	8.32±0.05	0.0±0.0	4.7	3.48±0.26	14.61±0.6	4.76±0.0	300.0±0.0	0.03 ± 0.0	7.56±0.07
**Mamry_au**	14.74±0.03	299±0.0	9.29±0.03	8.33±0.01	0.0±0.0	5.5	3.58±1.4	11.72±0.62	1.98±0.17	300.0±0.0	0.029±0.001	7.39±0.34

**Dargin_au**	15.28±0.03	309±0.0	9.90±0.08	8.33±0.01	1.4±1.57	4.5	6.13±0.13	26.97±0.91	11.81±0.46	333.33±57.74	0.026±0.001	7.78±0.05
**Kisajno_au**	15.21±0.01	316±0.0	9.01±0.01	8.32±0.02	0.33±0.06	3.3	12.35±0.88	27.37±1.21	7.34±0.46	300.0±0.0	0.037±0.0	8.23±0.27
**Niegocin_au**	15.28±0.02	376±0.0	10.05±0.05	8.37±0.01	2.27±0.32	2.6	9.51±0.89	62.97±1.13	39.71±0.86	300.0±0.0	0.038±0.003	8.93±0.18
**Boczne_au**	15.32±0.01	378±0.0	8.63±0.01	9.02±0.03	1.13±0.49	3.1	8.33±0.4	65.76±1.67	41.6±0.46	466.67±57.74	0.042±0.001	10.08±0.13
**Jagodne_au**	15.0±0.0	382±0.0	10.13±0.0	8.98±0.0	1.83±0.12	1.7	23.82±6.63	41.13±1.41	6.65±0.46	550.0±70.71	0.014±0.001	9.35±0.11
**Szymoneckie_au**	14.88±0.0	379±0.0	10.05±0.0	8.86±0.0	15. ±0.0	1.8	23.43±2.32	34.35±1.67	6.55±0.3	400.0±0.0	0.01±0.007	9.39±0.53
**Szymon_au**	14.070.0	414±0.0	10.12±0.0	8.43±0.0	3.0±0.0	1.1	18.84±0.98	36.94±0.62	2.78±0.17	633.33±57.74	0.013±0.0	11.33±0.19
**Tałtowisko_au**	14.78±0.01	388±0.0	10.55±0.01	8.68±0.01	0.47±0.06	1.8	17.37±1.92	20.99±0.91	2.48±0.34	300.0±0.0	0.016±0.0	9.58±0.09
**Tałty_au**	15.66±0.01	368±0.0	8.80±0.01	7.73±0.09	0.33±0.06	2.55	11.51±0.4	42.53±0.46	16.98±0.3	366.67±57.74	0.046±0.002	8.7±0.02
**Ryńskie_au**	15.18±	357.3±0.	9.50±0.0	7.77±0.12	0.63±0.06	2.15	16.9±0.9	36.94±1.	3.77±0.34	366.67±57.	0.031±0.003	9.18±0.03

	0.0	58						13		74		
**Mikołajskie_a**	15.82±0.01	350±0.0	10.01±0.01	7.67±0.03	0.73±0.06	2.4	15.41±0.43	39.04±0.62	8.54±0.34	300.0±0.0	0.030±0.001	8.57±0.07
**Bełdany_a**	15.33±0.0	316±0.0	9.99±0.0	7.76±0.04	1.8±0.0	1.7	16.47±0.54	44.02±1.13	9.33±0.17	300.0±0.0	0.029±0.001	6.97±0.06
**Śniardwy_a**	14.05±0.0	314±0.0	11.62±0.0	8.64±0.0	1.9±0.0	2.5	6.51±0.81	35.85±3.53	12.9±1.05	300.00±0.0	0.014±0.001	7.48±0.12

Temp – temperature; Cond – conductivity, SD - Secchi disc visibility, Chl a – chlorophyll *a* concentration; TP – total phosphorus; P-PO_4 – orthophosphates; TN – total nitrogen; NH_4^+ - ammonium; DOC – dissolved organic carbon; suffixes added to sampling locations (lake's names) indicate the studied seasons: _su – summer, _sp – spring, _au – autumn respectively

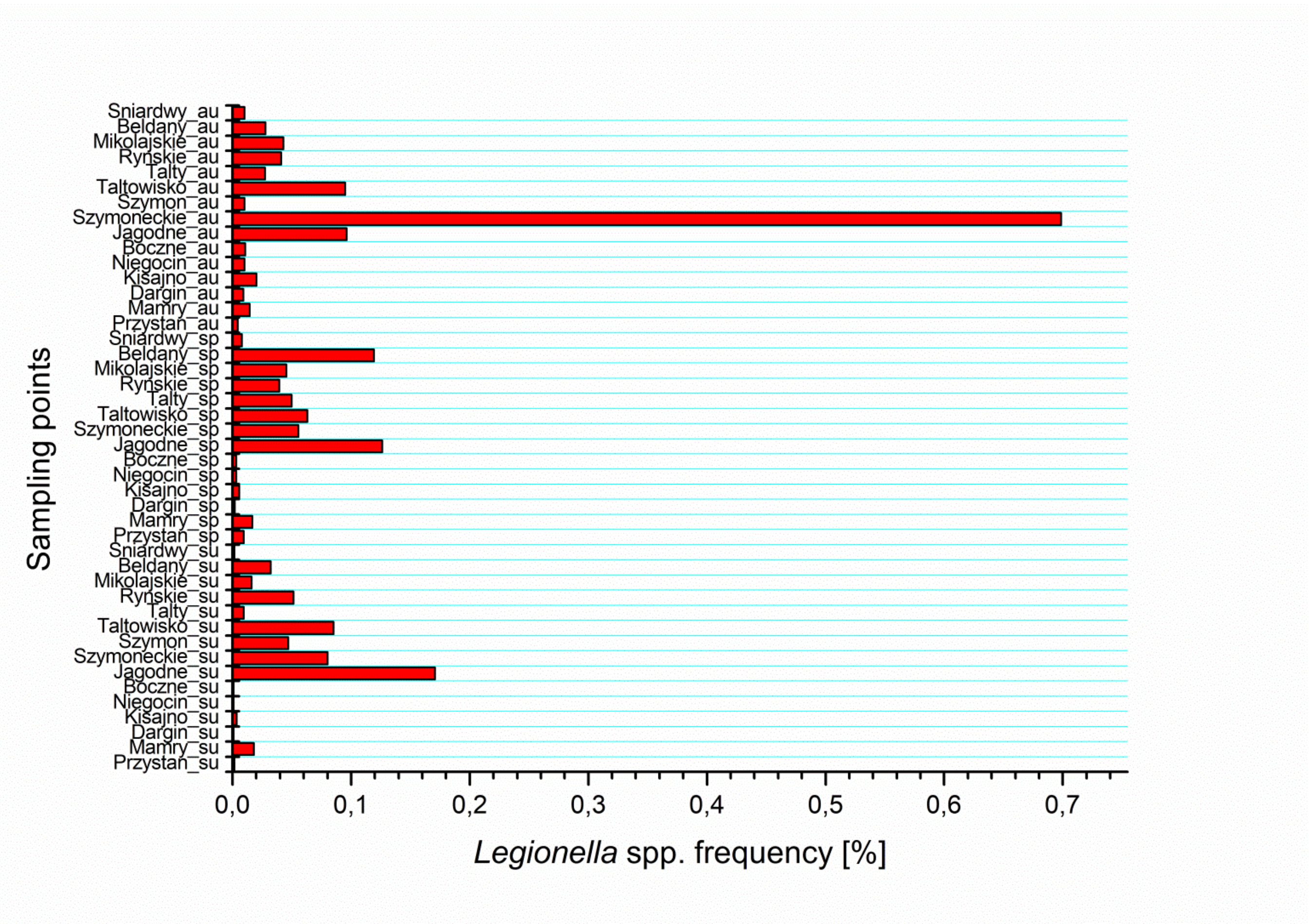

Figure S2 A. *Legionella* spp. relative abundance in all studied lakes.

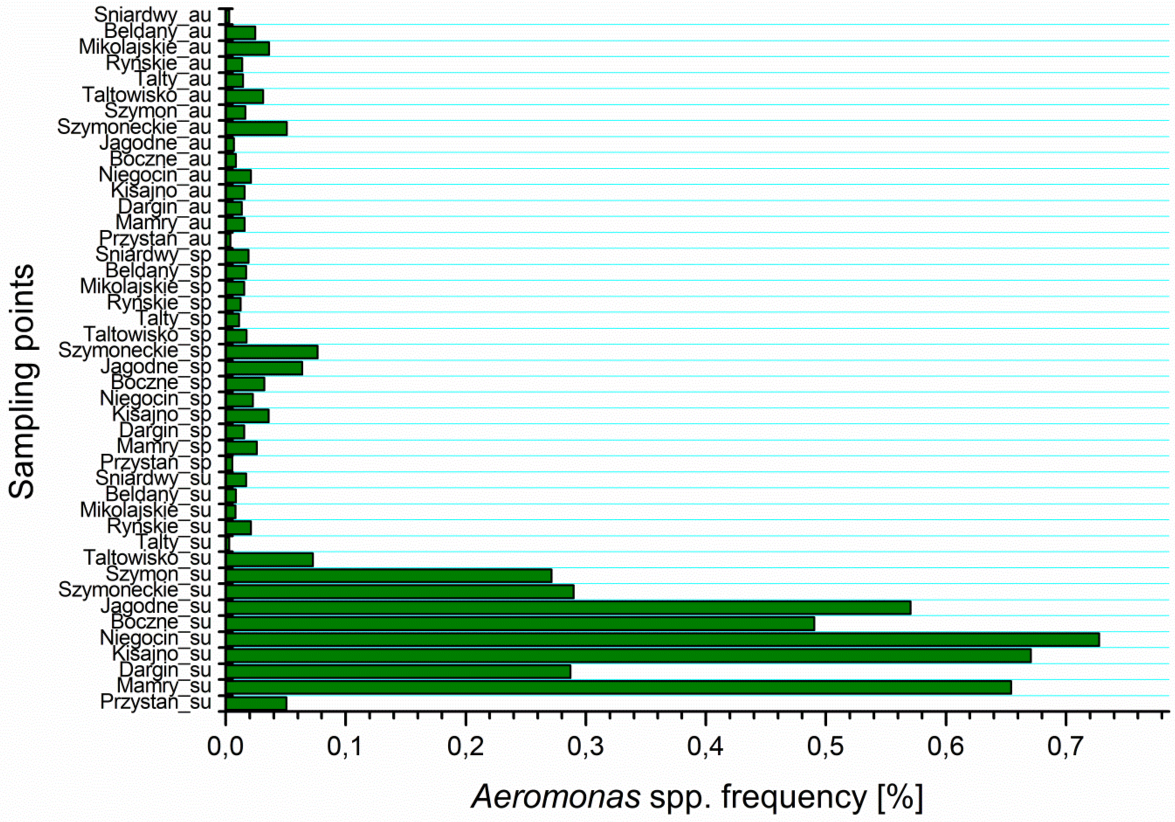

Figure S2 B. *Aeromonas* spp. relative abundance in all sampling points.

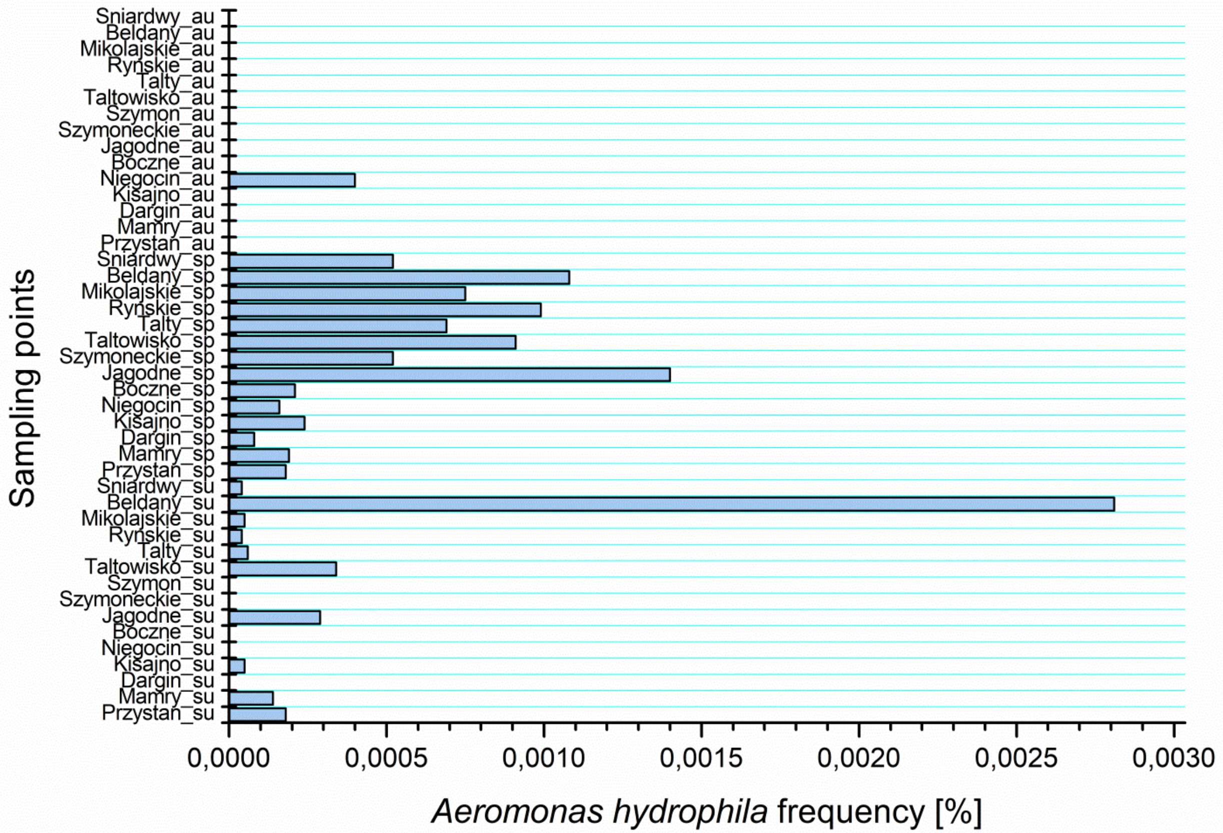

Figure S2 C. *Aeromonas hydrophila* relative abundance in all sampling points.
